# Unveiling amyotrophic lateral sclerosis complexity: insights from proteomics, metabolomics and microbiomics

**DOI:** 10.1093/braincomms/fcaf114

**Published:** 2025-03-19

**Authors:** Simone Scarcella, Lorenzo Brambilla, Lorenzo Quetti, Mafalda Rizzuti, Valentina Melzi, Noemi Galli, Luca Sali, Gianluca Costamagna, Giacomo Pietro Comi, Stefania Corti, Delia Gagliardi

**Affiliations:** Neuroscience Section, Department of Pathophysiology and Transplantation (DEPT), Dino Ferrari Centre, Università degli Studi di Milano, 20122 Milan, Italy; Neurology Unit, Foundation IRCCS Ca’ Granda Ospedale Maggiore Policlinico, 20122 Milan, Italy; Neurology Unit, Foundation IRCCS Ca’ Granda Ospedale Maggiore Policlinico, 20122 Milan, Italy; Neurology Unit, Foundation IRCCS Ca’ Granda Ospedale Maggiore Policlinico, 20122 Milan, Italy; Neurology Unit, Foundation IRCCS Ca’ Granda Ospedale Maggiore Policlinico, 20122 Milan, Italy; Neuroscience Section, Department of Pathophysiology and Transplantation (DEPT), Dino Ferrari Centre, Università degli Studi di Milano, 20122 Milan, Italy; Neurology Unit, Foundation IRCCS Ca’ Granda Ospedale Maggiore Policlinico, 20122 Milan, Italy; Neurology Unit, Foundation IRCCS Ca’ Granda Ospedale Maggiore Policlinico, 20122 Milan, Italy; Neuroscience Section, Department of Pathophysiology and Transplantation (DEPT), Dino Ferrari Centre, Università degli Studi di Milano, 20122 Milan, Italy; Neurology Unit, Foundation IRCCS Ca’ Granda Ospedale Maggiore Policlinico, 20122 Milan, Italy; Neuroscience Section, Department of Pathophysiology and Transplantation (DEPT), Dino Ferrari Centre, Università degli Studi di Milano, 20122 Milan, Italy; Neurology Unit, Foundation IRCCS Ca’ Granda Ospedale Maggiore Policlinico, 20122 Milan, Italy; Neuroscience Section, Department of Pathophysiology and Transplantation (DEPT), Dino Ferrari Centre, Università degli Studi di Milano, 20122 Milan, Italy; Neuromuscular and Rare Diseases Unit, Department of Neuroscience, Foundation IRCCS Ca’ Granda Ospedale Maggiore Policlinico, 20122 Milan, Italy; Neurology Unit, Foundation IRCCS Ca’ Granda Ospedale Maggiore Policlinico, 20122 Milan, Italy

**Keywords:** ALS, proteomics, metabolomics, microbiomics, lipidomics

## Abstract

Amyotrophic lateral sclerosis is the most common motor neuron disease and manifests as a clinically and genetically heterogeneous neurodegenerative disorder mainly affecting the motor systems. To date, despite promising results and accumulating knowledge on the pathomechanisms of amyotrophic lateral sclerosis, a specific disease-modifying treatment is still not available. *In vitro* and *in vivo* disease models coupled with multiomics techniques have helped elucidate the pathomechanisms underlying this disease. In particular, omics approaches are powerful tools for identifying new potential disease biomarkers that may be particularly useful for diagnosis, prognosis and assessment of treatment response. In turn, these findings could support physicians in stratifying patients into clinically relevant subgroups for the identification of the best therapeutic targets. Here, we provide a comprehensive review of the most relevant literature highlighting the importance of proteomics approaches in determining the role of pathogenic misfolded/aggregated proteins and the molecular mechanisms involved in the pathogenesis and progression of amyotrophic lateral sclerosis. In addition, we explored new findings arising from metabolomic and lipidomic studies, which can aid to elucidate the intricate metabolic alterations underlying amyotrophic lateral sclerosis pathology. Moreover, we integrated these insights with microbiomics data, providing a thorough understanding of the interplay between metabolic dysregulation and microbial dynamics in disease progression. Indeed, a greater integration of these multiomics data could lead to a deeper understanding of disease mechanisms, supporting the development of specific therapies for amyotrophic lateral sclerosis.

## Teaser

Scarcella *et al*. explore the role of multiomics in understanding amyotrophic lateral sclerosis, focusing on proteomics, metabolomics, lipidomics and microbiomics. They discuss how these approaches uncover pathogenic protein aggregation, metabolic alterations and microbial changes, which may lead to improved biomarkers, diagnosis and the development of targeted amyotrophic lateral sclerosis therapies.

## Graphical Abstract

**Graphical Abstract:**
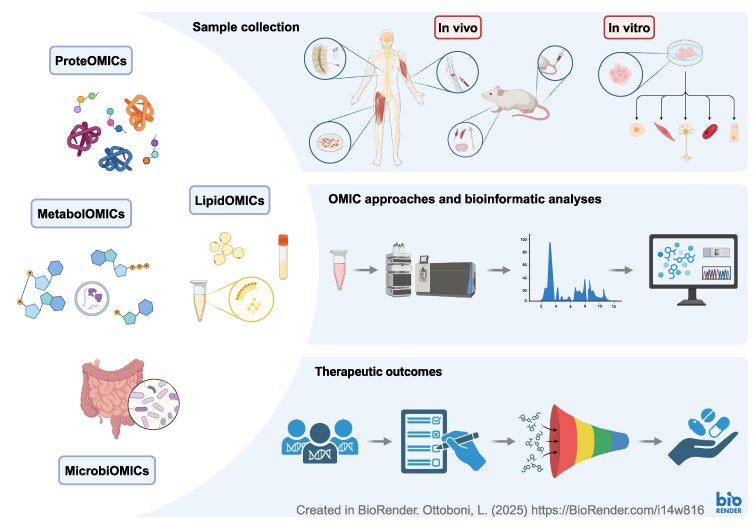

## Introduction

Over the last decade, the understanding of amyotrophic lateral sclerosis (ALS) has significantly evolved, with researchers shifting from viewing this condition as a singular disorder to recognizing it as a diverse spectrum of diseases. This change is attributed to the complexity intrinsic in its pathology. In addition to the progressive degeneration of motor neurons (MNs), patients with ALS may manifest with distinct clinical phenotypes, exhibiting different rates of disease progression and prognosis. The disease is mainly sporadic ALS (sALS), but up to 10% of cases are familial ALS (fALS). To date, more than 40 genes have been associated with sALS and fALS. The most common genetic causes of ALS are the hexanucleotide repeat expansion (HRE) in the chromosome 9 open reading frame 72 (*C9ORF72*) gene^1,2^ and mutations in superoxide dismutase 1 (*SOD1*)^3^ TAR DNA-binding protein 43 (*TARDBP*)^4^ or fused in sarcoma (*FUS*).^5,6^ Several biological and cellular pathways are implicated in the onset and progression of ALS, leading to pathophysiological heterogeneity. These pathomechanisms include defects in DNA repair, alterations in RNA metabolism, oxidative stress, neuroinflammation, mitochondrial dysfunction, the formation of insoluble protein aggregates, the alteration of vesicular transport, the dysregulation of autophagic processes and glutamate excitotoxicity.^7^

In the last decade, omic approaches have significantly advanced our understanding of ALS pathogenesis by identifying new pathogenic genes and common risk loci through genomic techniques.^8^ Together with genomics, transcriptomic analyses using advanced RNA-sequencing technologies have uncovered dysregulated molecular pathways, with RNA-targeted therapies offering potential treatments. The integration of multiomics data and technological innovations offers promise for better comprehension of ALS, improving patient stratification and developing targeted therapies. Indeed, the future of ALS treatment may lie in simultaneously targeting multiple molecular pathways.

The molecular and clinical heterogeneity of the disease could be responsible, at least in part, for the failure of the current clinical trials.^9^ Analysis of clinical data and biological samples via multiple high-throughput approaches may allow more precise stratification of disease subtypes and promote both biomarker discovery and personalized treatment development.

Large-scale genomic and transcriptomic studies have been instrumental to the development of personalized drugs by identifying genetic modifiers, pathways and potential therapeutic targets for complex diseases. Last year, a large multicentre study identified polygenic risk factors influencing symptom onset in a massive dataset of patients with *C9ORF72*-ALS/FTD.^10^ Integrating these insights, acamprosate, an FDA-approved GABA inhibitor, was prioritized and repurposed for its neuroprotective effects in *C9ORF72*-mutated *in vitro* models, showing similar efficacy to riluzole, the current standard of care. This study highlights the potential of genomics and transcriptomics in accelerating personalized therapies for neurodegenerative disorders.

In line with these findings, the integration of omics technologies has significantly advanced the understanding of ALS, pinpointing critical pathways, biomarkers and therapeutic targets for more accurate diagnosis and prognosis, suggesting that single omics approaches can struggle in fully capturing the complexities of ALS.

The combination of genomics, transcriptomics and other omic data has gained deeper insights into the disease molecular heterogeneity, paving the way for patient stratification and tailored therapies. Studies by Morello *et al*. demonstrated this concept, revealing two molecular subtypes of sALS through comprehensive profiling, which correlate with disease progression. They also identified histamine-related genes as potential biomarkers and therapeutic targets, with promising results from pharmacological modulation in experimental models.^11-15^ Together, these studies highlight the importance of integrating multiomics data to uncover ALS complex mechanisms and to develop more personalized treatment strategies.

In addition, mass spectrometry (MS), multiplex immunoassays and aptamer technologies can quantify the amount of proteins and transcripts in large groups of samples. These methods enable high-throughput analysis and offer deep insights into the molecular mechanisms underlying diseases such as ALS. To our knowledge, few proteomic studies have explored how protein aggregation, a hallmark of the disease, can be measured using innovative methodologies. Protein aggregation was recently quantified in lymphoblastoid cell lines from patients with ALS, revealing differences between sporadic and genetic forms.^16^ Specific drug candidates, including protein kinase inhibitors, reduced aggregation in sALS lymphoblasts but showed limited effects in genetic ALS, emphasizing disease heterogeneity. The study also explored proteome homeostasis in MNs, denoting variations in aggregation rates, which could aid personalized ALS drug discovery and evaluation. This approach promises a straightforward and customized method for disease characterization, as well as a valuable tool in ALS drug discovery and evaluation, emphasizing the need for tailored treatments based on individual patient profiles.^16^

Thus, the integration of these large-scale data through multiomics approaches may lead to the identification of complex pathways underlying the disease. Implementation of mathematics and statistics is fundamental to integrate big datasets and determine molecular patterns among the samples, and data interpretation might be critical. Combining omics with bioinformatics is essential to scale down the large number of potentially consistent molecules detected by omics to a set of clinically relevant candidates. Once candidates have been identified and a hypothesis has been formulated, targeted omics allow the validation of disease modifiers in larger cohorts.

Furthermore, given the wide phenotypic heterogeneity of ALS, a single biomarker is unlikely to encompass different disease subgroups or differentiate between patients and control subjects. Establishing panels of candidate altered and disease-relevant biomarkers is a possible strategy to provide specific signatures. This integrated approach may facilitate the categorization of clinically diverse patients into clearly defined subgroups, enabling the development of personalized medicine. The Answer ALS (AALS) programme has already collected demographic and clinical data from patients and multiomics findings from patient-derived induced pluripotent stem cells (iPSCs) and MNs, aiming at building an open source of integrated clinical and biological records.^17^ This programme offers a robust and high-powered tool to extract and analyse data from biologically relevant subgroups to further elucidate pathogenic mechanisms, identify molecular targets, and provide biomarkers to support the development of new therapeutic strategies.

**Figure 1:**
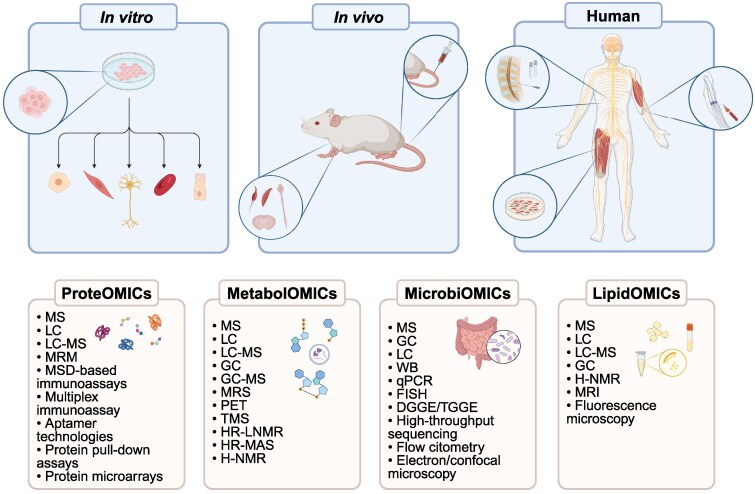
**Multiomics approaches for studying ALS.** Proteomics, metabolomics, lipidomics and microbiomics analyses are performed in *in vivo*, *in vitro* and human studies using a range of analytical techniques, including mass spectrometry, chromatography, molecular assays and imaging methods. WB, western blot; FISH, fluorescence *in situ* hybridization; DGGE/TGGE, denaturing/temperature-gradient gel electrophoresis (Created in BioRender. Ottoboni, L. (2025), https://BioRender.com/l17d202).

We have already provided an extensive overview of the literature on genomic, transcriptomic and epigenomic studies to investigate the pathomechanisms underlying ALS in detail.^8^ In this review, we summarize the major findings obtained from proteomic, metabolomic, microbiomic and lipidomic studies performed in patients as well as in *in vitro* and *in vivo* models of ALS and how the outcomes of large-scale studies have informed and facilitated more targeted validation studies (Fig. 1).

### Proteomics

Protein aggregation and inclusion formation are core features of ALS. Several studies have provided evidence supporting the role of various proteins in the pathomechanisms of ALS (Supplementary Table 1). In this context, the proteins most commonly associated with ALS include C9ORF72, SOD1, TDP-43 and FUS, which represent the main genetic causes of the disease. Notably, TDP-43 aggregates are commonly found in post-mortem CNS tissues from individuals with the vast majority of sporadic and familial forms of the disease. Conversely, cytoplasmic aggregates of FUS and SOD1 are present in the brain and spinal cord of patients with ALS harbouring *FUS* and *SOD1* mutations, respectively. Finally, dipeptide-repeat (DPR) proteins are *C9ORF72*-ALS-specific biomarkers, derived from non-canonical translation of repeated sequences-containing RNAs. Proteomic studies can help identify specific molecules that coaggregate or interact with these proteins, contributing to elucidate the molecular mechanisms underlying protein-protein aggregation and toxicity.^18^ Molecules with a strong significance could be probed with more targeted assays in large cohorts to investigate their predictive power in disease context. Interestingly, the identification of disease-related proteins in patient biofluids such as plasma, serum or CSF may also represent a reliable source of candidate biomarkers for several neurodegenerative disorders, including ALS. Moreover, these proteins may be used as potential pharmacodynamic markers to monitor treatment efficacy.

#### Studies on patients’ samples

TDP-43 inclusions represent the main pathological finding in most patients with sALS and fALS. A systematic review and meta-analysis from Majumder *et al.*^19^ showed that patients with ALS exhibit overall higher levels of CSF TDP-43 than both neurological and non-neurological controls do. Additionally, Ren’s group highlighted a correlation between CSF phosphorylated TDP-43 (pTDP-43) levels and disease severity measured by the ALS Functioning Disease Rating Scale-Revised (ALSFRS-R), as well as higher plasma levels of TDP-43 and pTDP-43 in patients than in healthy subjects.^20,21^ In a recent pilot study, Beyer’s group demonstrated that TDP-43 protein conversion to β-sheet-enriched structures was greater in CSF samples from patients with ALS than in those from patients with Parkinson’s disease and healthy controls.^22^ However, these findings still need to be confirmed with independent replications in larger cohorts.^23^ Furthermore, several post-transcriptional modifications of TDP-43, such as ubiquitination and hyperphosphorylation, have been reported to increase cytoplasmic and mitochondrial mislocalization and induce its aggregation. SarkoSpin, a technique that can distinguish between soluble and insoluble forms of disease-specific protein aggregates, allows the separation of physiological TDP-43 fractions from pathological inclusions.^24^ According to these studies, total and pTDP-43 plasma levels could reliably aid in the diagnosis of ALS, with very high sensitivity and specificity. Moreover, the detection of N-terminal truncated C-terminal fragments derived from TDP-43 pathological cleavage in human post-mortem brain tissues has facilitated the discrimination of patients with ALS from patients with other neurodegenerative diseases, including patients with Parkinson’s disease and Alzheimer’s disease.^25^ Taken together, these data suggest that TDP-43 correlates with disease severity and might be relevant for further investigations for differential diagnosis.^26-28^.

Furthermore, interactions between TDP-43 and many different proteins may be associated with the dysregulation of multiple biological processes. Indeed, Thompson’s group identified a specific subset of downregulated proteins in a cohort of patients with ALS and Parkinson’s disease compared with healthy subjects. Among these proteins, CSF dysregulation of putative elongation factor 1-alpha 1, histone H2B type 1-N, acidic leucine-rich nuclear phosphoprotein 32 family member A and Y-box-binding protein 1 (YBX1) was identified. Similarly, the expression of cytoskeletal components such as the tubulin beta chain was downregulated. The expression levels of synaptic proteins (neurexin 1 and neurexin 3), neural growth factors (ephrin type A receptor 4), guidance proteins and cell adhesion molecules (cadherin 13 precursors) were also decreased, reflecting underlying synapse loss.^29^

The pathogenic HRE in *C9ORF72* is the most common genetic cause of ALS in the Caucasian population. *C9ORF72*-related ALS is neuropathologically characterized by the presence of TDP-43-negative, ubiquitin- and p62-positive and intranuclear and cytoplasmic neuronal inclusions that contain DPR proteins.^30^ DPRs are peptides derived from unconventional repeat-associated non-ATG translation of repeated RNA foci transcribed from the HRE and represent *C9ORF72*-ALS biomarkers.^31,32^ Different types of DPRs can be isolated via chromatography and quantified via Meso Scale Discovery-based immunoassays. Indeed, both sense and antisense transcription can occur with the subsequent generation of five types of DPRs, among which poly-glycine–proline levels in patient CSF may be negatively correlated with the size of the HRE. Conversely, no association has been demonstrated with the disease progression rate.^27,33^

SOD1 protein in the CSF of patients presenting mutations in the *SOD1* gene and treated with *SOD1*-directed antisense oligonucleotides (ASOs) has been employed as a pharmacodynamic biomarker to quantify target engagement in clinical trials.^34,35^ Indeed, this protein is detectable in the CSF of patients with ALS harbouring a mutation in *SOD1*, and the protein expression was successfully decreased after the administration of ASOs targeting *SOD1* in both mouse models and clinical trials.^35-38^ To date, no clear evidence concerning the relationship between the levels of misfolded SOD1 in the CSF of patients and disease progression has been reported.^39^ In the VALOR Phase III clinical trial (NCT02623699), treatment with tofersen, a *SOD1*-directed ASO, led to a reduction in SOD1 levels in the CSF of a faster-progression subgroup of patients, with no significant effect on modulating the decline in the ALSFRS-R score.^37^

Neurofilaments (NFs) are fundamental neuronal cytoskeletal components whose degree of damage may be correlated with the degree of axonal loss underlying neurodegenerative diseases, such as MNDs, and, more specifically, ALS.^40^ Increased levels of neurofilament light chain (NFL) and phosphorylated neurofilament heavy chain (p-NFH) in the CSF and plasma samples of patients with ALS have been associated with a shorter life expectancy and more rapid disease progression.^27,41^ While NFL levels seem to be correlated with disease stage, speed of progression and survival, no clear association has been established with the ALS phenotype.^26,28^ Conversely, pNFH levels are reportedly higher in the CSF of patients with *C9ORF72*-ALS than in that of patients with sALS.^42,43^ Given their stability over time, measurements of blood NFs might be applied in pharmacodynamic monitoring, as NF levels in CSF have been shown to be reduced by *SOD1*-directed ASO treatment (VALOR, NCT02623699).^37^

In addition, the levels of markers of microglial activity and neuroinflammation may be increased in individuals with ALS. Among these proteins, chitotriosidase 1 (CHIT1), chitinase-3-like protein 1 (CHI3L1/YKL40) and chitinase-3-like protein 2 (CHI3L2/YKL39) are the most relevant.^29,44^ Despite their lower diagnostic accuracy, these markers may still have diagnostic value, particularly when NFs are used as reference biomarkers.^27,44^ Evidence indicates the ability of these biomarkers to correlate with disease severity, progression rate and survival.^28,45,46^ In a cross-sectional analysis, these analytes were found at low concentrations in the CSF of asymptomatic carriers, with their levels increasing with symptom onset, providing a strong rationale for their use as candidate diagnostic biomarkers.^28,47^ Conversely, in another study, patients harbouring *C9ORF72* and *SOD1* mutations expressed increased levels of CHIT1 in the presymptomatic phase, suggesting that an inflammatory process is already active before the onset of clinical symptoms.^48^ CHIT1 and CHI3L2 were also increased in patients with ALS compared with patients with primary lateral sclerosis (PLS), whereas CHI3L1 levels were similar in patients with both ALS and PLS. This outcome was limited to a small number of subjects and still needs validation in a larger cohort; however, if confirmed, chitinases could help in the differential diagnosis between PLS and ALS.^44^

Our group has recently investigated the CSF levels of NFL, CHIT1 and miR-181b in a large multicentre cohort of patients with ALS and control patients,^49^ showing that NFL has the most effective diagnostic performance and is the best independent predictor of disease progression and survival. Interestingly, all three biomarker levels were increased compared to controls affected by neurodegenerative diseases.

Barschke *et al.* reported increased CSF levels of NFM and CHIT1 in patients with *C9ORF72*-ALS, possibly reflecting neuroinflammation and neurodegeneration.^50^ In addition, these researchers showed that CHI3L2 CSF levels were increased in *C9ORF72*-ALS but not in *C9ORF72*-FTD, suggesting potentially different roles of microglia in the pathophysiology of these diseases. According to their findings, *C9ORF72*-ALS samples presented upregulated UCHL1, highlighting the relevance of ubiquitination and autophagy in disease pathogenesis. Conversely, the researchers reported a downregulation of neuronal pentraxin receptor, a synaptic transmembrane protein that plays a crucial role in synaptic organization through its interactions with other pentraxin proteins, in the CSF of patients with *C9ORF72*-FTD. Thus, its downregulation might be an expression of synaptic dysfunction in FTD.^50^

Monocyte chemoattractant protein 1 (MCP-1), also known as C–C motif chemokine ligand 2 (CCL2), is a neuroinflammatory mediator that is present at relatively high levels in the CSF of patients with ALS, reflecting disease severity, as measured by the ALSFRS-R, but has no clear association with disease duration.^51^ However, MCP-1 concentrations have been associated with disease progression and survival in patients with *C9ORF72*-ALS.^28,43,52^ Similarly, higher concentrations of macrophage inflammatory protein 1α (MIP-1α) and 1β (MIP-1β), also called CCL3 and CCL4, in the CSF of patients with ALS have been reported to correlate with slower disease progression and longer survival.^28,43,53^

Among chemokines, granulocyte colony-stimulating factor and interleukin-9 (IL-9) are predictors of longer survival, whereas IL-5, IL-12 and IL-8 are associated with shorter lifespan. IL-8 has been shown to be positively correlated with disease severity. An increase in the levels of IL-15, IL-18, IL-4 and CCL11 has been linked with fast-progressing ALS, whereas an increase in the level of IL-10 has been reported to be associated with lower scores on the ALSFRS-R.^28,54-57^. The tumour necrosis factor α concentration in the CSF has been suggested to be a predictive factor of shorter life expectancy in patients with *C9ORF72*-ALS.^28,58^

Oeckl *et al.* performed a CSF proteomic study through liquid chromatography coupled with mass spectrometry (LC-MS) and multiple reaction monitoring in mutation carriers with asymptomatic and symptomatic ALS and patients with sALS, as well as in post-mortem spinal cord tissues of patients with ALS and control individuals. These researchers confirmed changes in the NFL, NFM, NFH, CHIT1 and CHI3L1 protein levels and identified novel differentially expressed proteins (DEPs), including ubiquitin C-terminal hydrolase-L1 (UCHL1), microtubule-associated protein 2 (MAP2), capping actin protein, gelsolin-like (CAPG) and glycoprotein non-metastatic melanoma protein B (GPNMB).^47^ UCHL1 dissociates ubiquitin from small peptides and plays a role in the homeostasis of a functional ubiquitin-proteasome system (UPS). UCHL1 is crucial for axonal function, and its absence leads to corticospinal and MN degeneration. The increased levels of UCHL1 in the CSF of patients with ALS might reflect its release as a consequence of axonal degeneration, whereas its reduction in post-mortem tissue could be secondary to axonal loss.^47^

Other candidate diagnostic biomarkers measured by Morello *et al.* include IL-10, IL-6, GM-CSF, IL-2 and IL-15, while altered protein levels of MIP-1α, wrCRP, HMGB, creatine kinase, granzyme B, IL-8, cystatin C, GPNMB, UCHL1, bFGF and NGF show potential as prognostic indicators rather than diagnostic biomarkers.^27,41^

Overall, NFs and neuroinflammatory molecules (CHIT1, CHI3L1 and CHI3L2) are reliable biomarkers for diagnosis and are correlated with disease severity, rate of progression and survival.^27,28,40,41^

As one of the principal microtubule-associated proteins in neurons, tau protein orchestrates the regulation of cytoskeletal stability. In pathological conditions, tau can form intracytoplasmic and axonal toxic aggregates, leading to neurodegeneration. Different studies have investigated the role of tau and its phosphorylated form (pTau) in ALS. Despite being controversial, the results suggest the relevance of pTau in the differential diagnosis of ALS, although with less accuracy than that of NFs.^59^ In addition, the pTau/tTau (total tau) ratio has been proposed to be correlated with disease progression and survival, as well as severity, as measured by the ALSFRS-R, indicating its potential as a prognostic biomarker.^28,60^ In fact, the pTau/tTau ratio may be clinically relevant since a reduced ratio is associated with MRI signs of corticospinal tract and grey matter atrophy, as well as upper MN (UMN)-dominant ALS phenotypes.^28,59-64^. A promising and recently emerged biomarker for ALS might be tau phosphorylated at threonine 181 (p-Tau181), which is present at higher levels in the plasma of patients with ALS than in that of healthy controls. p-Tau181 elevation in plasma seems to be particularly associated with lower motor neuron (LMN) loss.^65-67^. However, subsequent studies have debated these outcomes, with some analyses reporting no differences in CSF tau levels between patients with ALS and healthy subjects, thus highlighting the need for additional prospective biomarker studies.^26,27^

Even with contradictory evidence, p-Tau can also help in diagnosis, with the p-Tau/t-Tau ratio correlating with some clinical features.^28,59,60^ MAP2 belongs to the same family of tau, but it is restricted to the somatodendritic compartment, whereas tau is more represented in the axons. MAP2 may be overexpressed in the CSF of patients with ALS.^47^ CAPG and GPNMB are associated with inflammation and exhibit increased levels in the CSF and spinal cord tissue of patients with ALS.^47^ Specifically, increased levels of GPNMB have been associated with a shorter life expectancy and greater disease severity, as assessed by the ALSFRS-R.

Altered levels of RNA-binding motif 45 (RBM45) protein have been reported in the CSF and spinal cord of patients with ALS.^68^ This protein shares sequences with TDP-43 and FUS, with the ability to precipitate and form intracytoplasmic inclusions, which were found in 91% of the patients with ALS in this study. Moreover, the dysregulation of fat metabolism may be associated with ALS since patients may present increased levels of ApoE in the plasma, more rapid disease progression and shorter survival.^69^ Finally, several other proteins are reported to exhibit altered levels among patients with ALS, but whether these alterations have clinical relevance still needs to be assessed.

#### *In vivo* studies

Leveraging proteomic studies in *in vivo* models, such as *Mus musculus* (mouse), *Danio rerio* (zebrafish), *Drosophila melanogaster* and *Caenorhabditis elegans*, has proven pivotal in elucidating disease-related pathways and identifying potential therapeutic targets.

Particularly, murine models have been crucial in ALS research, as their genetic manipulability enables the modelling of specific mutations, making them an invaluable tool for exploring the molecular mechanisms underlying ALS. Proteomic studies in SOD1-G93A transgenic mice have revealed critical insights into the molecular alterations occurring during ALS onset and progression. Notably, a group of 189 DEPs have been recently identified in the spinal cord of SOD1-G93A mice at disease onset.^70^ Beyond multiple pathways identified, several of these proteins were associated with immunity and inflammation, supporting their critical role at the early disease stage of ALS.^70^ Xu *et al.* expanded upon this research by integrating proteomic and metabolomic data, offering a more comprehensive understanding of the biological processes involved. Their analysis uncovered significant dysregulation in several critical metabolic pathways, including (i) purine metabolism, which plays a key role in nucleotide synthesis and energy transfer, (ii) methionine cycle, a central component of methylation reactions and sulphur amino acid metabolism, and (iii) fatty acid metabolism, which is essential for energy production, cellular signalling and membrane synthesis. This integrative approach provided deeper insights into the complex interplay of these pathways in the context of the studied condition.^71^ In a previous study, quantitative multiplexed proteomic analysis was employed to investigate the role of ubiquilin2 (UBQLN2) in regulating proteome composition in several Ubqln2-based murine models of ALS.^72^ UBQLN2, a ubiquitin receptor, binds to the proteasome through its ubiquitin-like domain, facilitating the delivery of ubiquitinated proteins for degradation. Mutations in *Ubqln2* are associated with fALS and FTD. In this study, the authors discovered that UBQLN2 dysfunction leads to proteomic changes that affect multiple pathways, with serotonergic signalling being particularly impacted. Notably, they observed an upregulation of proteasome subunits, suggesting a compensatory response to diminished proteasome activity. Among the proteins whose abundance is linked to UBQLN2 function, the most significant were the ubiquitin ligase TRIM32 and two retroelement-derived proteins, PEG10 and CXX1B, offering new insights into proteins whose degradation is regulated by UBQLN2.^72^

Zebrafish have been widely employed as a model organism in the study of neurodegenerative diseases, including ALS. Frøyset *et al*. exposed zebrafish embryos to sub-lethal doses of the neurotoxin β-methylamino-L-alanine (BMAA), a compound previously linked to ALS pathology. While BMAA exposure did not induce developmental abnormalities, it resulted in a reduced heart rate and upregulation of GSK3 isoforms. Proteomic analysis revealed the dysregulation of proteins involved in glutamate receptor signalling, protein homeostasis and reactive oxygen species (ROS) generation. These findings underline the utility of zebrafish in exploring neurotoxic mechanisms relevant to ALS.^73^

The *D. melanogaster* model has proven to be a powerful *in vivo* system for exploring neurological disorders, including ALS. The group of Velentzas employed a liquid chromatography–tandem mass spectrometry technology to map the proteomic landscape of the fly brain, identifying thousands of proteins associated with signalling pathways and neuronal diseases. Their work highlighted the role of the UPS in neuronal function, showing that disruption of UPS components affects longevity and locomotor activity. This study underscores the importance of proteostasis in ALS and related neurodegenerative disorders.^74^

The *C. elegans* model has emerged as a valuable tool for studying ALS-related genes, including those associated with the *C9ORF72* HRE, as demonstrated by its use to investigate neurodegeneration and toxic DPR production.^75^ Concerning proteomic studies, *C. elegans* has been employed to evaluate the impact of these genetic mutations on cellular stress responses, metabolism and protein homeostasis. Indeed, Elsayyid *et al.* introduced a novel on-filter in-cell processing method for proteomic analysis, enabling the identification of over 9400 proteins from just 200 worms through single-shot LC–MS analysis. This streamlined approach was used to investigate the effects of *sod-1* loss, revealing changes in proteins linked to stress response and metabolism. Given its flexibility and high sensitivity, this approach holds promise for broader applications in other model organisms and systems, making it an invaluable tool for exploring genetic perturbations and their effects on cellular processes.^76^

Overall, *in vivo* models offer valuable platforms for exploring proteomic alterations in ALS and identifying potential pathways for therapeutic intervention, paving the way for new opportunities in drug discovery and biomarker identification.

#### *In vitro* studies

*In vitro* proteomic studies have become pivotal in unravelling the molecular mechanisms underlying ALS. Patient-specific cellular models, such as patient-derived fibroblasts and iPSC-derived MNs, and primary cultures, provide valuable platforms for identifying protein alterations and deciphering their role in disease progression. These studies have elucidated disruption in critical cellular pathways, including proteostasis, intracellular transport, mitochondrial function and cytoskeletal integrity, all of which are central to ALS pathogenesis.

In the last decade, proteomic studies performed on different *in vitro* models, have highlighted microglial dysfunction and mitochondrial impairment as key contributors in disease pathogenesis,^77-80^ demonstrated cryptic exons translation into new peptides in TDP-43-depleted cellular models,^81^ and shed light on defects in cellular transport mechanisms^82,83^ and protein interaction networks through advanced interactomics,^36,84,85^ contributing to increase our understanding on ALS.

Using cultured microglia derived from symptomatic SOD1-G93A mice, temporal proteomic and functional changes associated with ALS progression were explored,^77^ identifying two distinct molecular profiles at early and advanced disease stages. While proteins linked with microglial function, such as GPNMB and HMBOX1, were upregulated in early stages, rootletin, major vaults proteins and STK38, linked to RNA metabolism, emerged in later stages. These results suggest that, over the progression of the disease, chronically activated microglia progressively lose their immune features.

Other studies emphasized the role of mitochondrial dysfunction in ALS progression, uncovering complex neuroinflammatory interactions between iPSC-derived astrocytes and microglia, and focusing on alterations in mitochondrial proteins, including voltage-dependent anion channels and components of the respiratory chain, linking energy metabolism defects to neuronal vulnerability.^78,79^ Lin *et al.* investigated the contribution of the ALS-associated UBQLN2 P497S mutation in mitochondrial impairment, adding evidence that mitochondrial abnormalities are closely linked to disease pathogenesis in ALS. Mitochondrial dynamics were disrupted in UBQLN2 mutated-mouse model and in knockout (KO) cells, suggesting that the loss of UBQLN2 may compromise mitochondrial health by interfering with protein import and regulation, focusing on the reduction of the TIMM44 protein, which is essential for mitochondrial protein translocation.^80^

Using *SOD1*-mutated iPSC-derived MNs, Tsioras *et al*. studied several proteins involved in folding and cytoskeletal homeostasis, including the ALS-causing valosin-containing protein (VCP), involved in protein trafficking and degradation. They showed a slower VCP turnover in *SOD1*-mutated models, compared with isogenic controls, resulting in VCP accumulation and alterations in its interactome. Therefore, mutations in VCP may exacerbate SOD1 toxicity by disrupting protein quality control and its modulation could offer a therapeutic target for patients with *SOD1*-mutated ALS.^86^

In addition, Une *et al.*^36^ highlighted the role of misfolded SOD1 aggregates, revealing that proteolytic dysfunction and alterations in cytoskeletal proteins, such as actin and tubulin, contribute to ALS progression.

In parallel, the application of both transcriptomic and proteomic analyses on TDP-43-depleted iPSC-derived neurons shed new important insights into cryptic splicing. Indeed, cryptic exons generated by mis-splicing due to TDP-43 loss of function are not only able to inhibit the expression of mRNA transcript through promoting their degradation, but have been shown to induce the translation of *de novo* proteins from pathological transcripts.^81^ Since TDP-43 depletion *in vitro* resembles splicing abnormalities observed in ALS/FTD brains, these findings may offer a promising approach for assessing TDP-43 activity in patient CSF and point towards novel mechanisms underlying ALS/FTD pathophysiology. Conversely, Atkinson *et al*.^85^ examined the effects of TDP-43 overexpression on cultured neuronal cytoskeleton dynamics, revealing that increased TDP-43 promotes branching in heterozygous neurons but leads to toxicity and aggregation in homozygous neurons, underlining the critical need for precise regulation of TDP-43 levels.

Protein quality control mechanisms and a dysregulation in pathways involving mitochondrial dysfunction and impaired ATP synthesis were investigated in fibroblasts from patients with *C9ORF72* HRE through MS-based proteomic analysis.^87^ These disruptions in proteostasis, including the downregulation of key chaperones such as HSP90α and BiP, exacerbate ALS susceptibility and may contribute to the generation of cryptic peptides, as seen in TDP-43 depletion and mis-splicing.^88^ Similarly, Narayan *et al*.^89^ identified altered protein expression linked to protein folding and stress responses in ALS fibroblasts. Additionally, the accumulation of Poly(GP) in *C9ORF72*-associated ALS highlights the broader theme of misfolded proteins in ALS, with its presence serving as a biomarker for disease progression.^27,34,42,90^ Both Poly(GP) and cryptic peptides offer potential diagnostic and therapeutic targets, as monitoring their levels may provide insights into disease progression and treatment efficacy. These findings collectively emphasize how disrupted protein degradation and misfolding contribute to ALS pathophysiology, reinforcing the need for targeted therapies to address proteostasis and mitigate neurodegeneration.

RNA transport is a critical process for maintaining cellular function and homeostasis, and its disruption has been implicated in a range of neurodegenerative diseases, including ALS. Kim *et al.*^82^, utilizing quantitative MS and RNA-seq, investigate how ALS mutations alter the nucleocytoplasmic distribution of proteins and transcripts and provide a comprehensive dataset of the nucleocytoplasmic distribution of the proteins and transcripts. The results from this work indicate that RNA transport proteins are predominantly shifted to the nucleus in SOD1-G93A mutant cells, suggesting that defects in RNA transport may contribute to ALS pathology.^82^ Another study focuses on RNA granule transport, highlighting the role of lysosomes and the RNA-binding protein ANXA11 in facilitating long-distance RNA transport in neurons. ALS-associated mutations in *ANXA11* impair this process, leading to deficits in axonal RNA transport and neuronal dysfunction.^83^

Interactomics, the study of protein–protein interactions, plays a pivotal role in understanding the molecular mechanisms of ALS. Many studies have employed advanced techniques such as MS and protein pull-down assays to unravel the interactomes of ALS-associated proteins, providing valuable insights into disease pathophysiology. One of the above-mentioned studies delves into the interactomes of six ALS-associated proteins, including ATXN2, FUS, TDP-43, OPTN and UBQLN2, highlighting the common cellular pathways they regulate.^84^ Notably, ATXN2, FUS and TDP-43 were found to share interactors that regulate MN function through similar mechanisms, suggesting a convergence of pathogenic pathways in ALS. This study also revealed that OPTN and UBQLN2 interact with autophagy-related proteins and those involved in NF-κB signalling. Moreover, FMRP, identified as a shared interactor between ATXN2, FUS and TDP-43, was proposed as a therapeutic target, particularly in FUS-related toxicity.^84^

Collectively, all these findings underline the multifaceted nature of proteomic dysregulation in ALS. *In vitro* studies provide a crucial foundation for understanding these complex pathways, identifying biomarkers and uncovering therapeutic targets. As proteomic technologies advance, such models are poised to further elucidate ALS pathology and inform precision medicine approaches.

### Metabolomics

Metabolomics refers to the global search for metabolites, generally recognized as the downstream products of the biological processes carried out in cells, tissues and biological fluids. Different studies on cell-based assays, animal models and human samples have identified different metabolites and specific metabolic signatures involved in human diseases. Overall, findings from studies performed in patients and animal models suggest that metabolic alterations in ALS may follow a spatiotemporal pattern, especially in *in vivo* disease models. Nevertheless, definitive evidence that associates a specific metabolic fingerprint with ALS is still lacking.

#### Human studies

Early non-invasive metabolomic approaches for ALS were based on *in vivo* magnetic resonance spectroscopy (MRS) to measure brain metabolites.^91,92^ These neurochemical changes might be helpful in the longitudinal monitoring of ALS disease progression.^93,94^

Notably, different brain regions presented various metabolic profiles. For example, the motor cortex of patients with ALS presented decreased N-acetylaspartate (NAA) levels, suggesting that neuronal loss in this area is correlated with disease severity and impaired executive functions.^91,95-97^. MRS imaging analysis of the brainstem, thalamus, posterior cortex and cerebellum revealed similar results, with the degree of reduced NAA being related to the severity of UMN abnormalities.^91,98-100^. MRS analysis of the cervical spinal cord of patients with ALS revealed a significant reduction in the NAA/creatinine (Cr) and NAA/Myo-inositol (Myo) ratios associated with disease progression.^101,102^ Notably, asymptomatic subjects carrying *SOD1* mutations present analogous findings, indicating that these metabolic changes may precede disease onset.^103^

Different lines of research suggest that ALS might be associated with an imbalance between excitatory neurotransmitters, as measured by glutamate, and inhibitory neurotransmitters, as assessed by gamma-aminobutyric acid (GABA).^104,105^ Although the precise pharmacological mechanisms *in vivo* are still unknown, riluzole is one of the few approved medications for ALS able to modulate excitatory neurotransmission. The use of the MRS technique to evaluate *in vivo* brain metabolite profiles of riluzole-treated versus riluzole treatment-naive patients with ALS revealed that the former presented reduced glutamine (Gln) levels compared with those in untreated patients with ALS and healthy controls, indicating a potential role of Gln as a clinically relevant marker of disease.^105^ Riluzole might increase the NAA/Cr ratio in the motor cortex over time, indicating the presence of a population of metabolically dysfunctional neurons amenable to treatment.^106^

A recent study using 18F-fluorodeoxyglucose PET in presymptomatic *C9ORF72* HRE carriers revealed the presence of clusters of hypometabolic brain areas compared with healthy controls. These alterations preceded the increase of NF levels in biofluids and the onset of symptoms, suggesting a potential role of PET as a sensitive biomarker in a presymptomatic phase in this subgroup.^107^

Taken together, these findings highlight the potential contributions of PET and MRS in defining several metabolic hallmarks in patients with ALS *in vivo*, as well as in evaluating disease progression and response to treatment.

Cortical hyperexcitability, assessed by transcranial magnetic stimulation (TMS), is a valuable indicator of UMN dysfunction in individuals with ALS.^108,109^ Single-pulse TMS studies revealed an early reduction in the resting motor threshold in patients with ALS, which is indicative of cortical hyperexcitability, followed by a progressive increase as the disease progresses, eventually leading to inexcitability.^108-111^ Approximately 20% of patients with ALS exhibit inexcitability of the motor cortex, typically occurring at later stages;^109,112^ conversely, cortical inexcitability is more commonly observed in patients with PLS.^112^ Central motor conduction time (CMCT) prolongation is consistently observed in individuals with ALS, likely due to desynchronization of corticomotoneuronal transmission caused by corticomotoneuronal fibre degeneration.^108,109,113^ Prolonged CMCT can reveal the involvement of the corticospinal tract in patients with ALS with muscle atrophy due to spinal MN impairment. Additionally, detecting corticomotor neuron loss in individuals with ALS, even in subclinical stages, can help differentiate between central and lower MN degeneration.^108,109,114^

The duration of the cortical silent period is consistently shortened in patients with ALS across all phenotypes, reflecting a combination of inhibitory interneuron degeneration and dysfunction in GABA B-mediated receptor inhibition.^108,109,115^

Studies using paired-pulse TMS have identified cortical hyperexcitability as a key mechanism in ALS. This process involves a combination of decreased cortical inhibition and increased cortical facilitation, which are recognizable neurophysiological characteristics of the disease. The absence or reduction of short-interval intracortical inhibition (SICI) has been recognized as an early feature of ALS and serves as an adverse prognostic biomarker. In patients with ALS, a reduced SICI is accompanied by increased intracortical facilitation, indicating increased cortical excitability, which might be potentially helpful in distinguishing ALS from other neuromuscular mimicking disorders.^108,109^

The transcranial stimulation threshold complements standard neurophysiological assessments and increases diagnostic precision in ALS. Moreover, treatment with riluzole may transiently increase SICI, with possible utility as a pharmacodynamic biomarker.^108,109^

Although they are valuable for defining the metabolic signature of ALS, these neurochemical changes are non-specific for the disease, since they may overlap with different disorders involving impaired neuronal integrity, such as brain tumours, ischaemia and trauma.^116^ This reduced specificity depends on the limited number of metabolites that can be assessed in humans *in vivo*.

The analysis of human body fluids, such as blood, urine and CSF, provides an alternative approach to enable a more comprehensive investigation of the ALS metabolome through techniques such as high-resolution liquid nuclear magnetic resonance, high-resolution magic angle spinning and MS.

High-performance liquid chromatography has also been employed to analyse the plasma of 28 patients with ALS compared with that of 30 healthy controls.^117^ The metabolic profile of patients with ALS is characterized by a decrease in certain metabolites. In particular, patients exhibiting a prevalent LMN phenotype presented a distinct set of differentially expressed metabolites. Furthermore, the study revealed a significant increase in the levels of 12 molecules among patients receiving riluzole. Despite these promising results, these preliminary findings require further validation in larger cohorts. A similar study on patients with possible, probable and definite ALS, according to the revised El Escorial criteria,^118^ identified numerous significantly altered compounds in the plasma from patients with ALS that were associated with hypermetabolic processes, oxidative changes and mitochondrial dysfunction.^119^

Similarly, metabolomic profiling of blood samples from recently diagnosed patients with ALS compared with those from patients with ALS-mimic syndromes and healthy controls revealed a subset of several candidate metabolites that cosegregated with these diagnostic groups with high sensitivity (90%), suggesting the effectiveness of biochemical profiling in distinguishing ALS from disease mimics.^120^ In particular, creatinine levels showed the strongest correlation with lower ALSFRS-R scores and outcomes, as confirmed by another study in which extensive blood testing was performed in more than 700 patients with ALS.^120,121^ Despite these valuable findings, metabolomic profiles in blood samples collected years before symptom onset do not reliably distinguish patients with presymptomatic ALS from controls.^122,123^

Other studies focusing on specific metabolites in the serum of patients with ALS highlighted altered levels of homocysteine, NAA and urate, whereas branched-chain amino acids measured via LC-MS in prediagnostic individuals might not be associated with the risk of ALS.^124-127^

Metabolomic analysis can be used in combination with pharmacological approaches to assess the biological effects of investigational drugs. A targeted metabolomics approach using tandem MS on plasma from patients with ALS treated with olesoxime, a compound with neuroprotective properties, was used to discriminate between treated and control groups. Specifically, glycine, kynurenine and citrulline/arginine are more reliable predictors of group classification. In addition, Gln levels and molecules linked to lipid metabolism are correlated with clinical progression.^128^

Blasco *et al*. quantified diverse metabolites in the CSF of patients with ALS, revealing decreased levels of acetate and increased concentrations of pyruvate and ascorbate. This metabolic profile accurately classified the patient group in most cases.^129^ In addition, CSF analysis via gas chromatography (GC) and LC-MS revealed alterations in energy utilization pathways. In particular, glucose, α-hydroxybutyrate, leucine, isoleucine and ketoleucine, which are valuable potential biomarkers, are increased in patients with ALS.^130^ In a longitudinal study performed on CSF through proton nuclear magnetic resonance (H-NMR), increased levels of glucose, lactate, citric acid and ethanol were observed in patients with ALS, supporting the role of a hypercatabolic state in this disease.^131^ Wuolikainen *et al.* analysed the metabolome signatures of 120 compounds in the CSF of patients with ALS via GC coupled to time-of-flight mass spectrometry. In this study, patients were categorized according to their hereditary background and clinical subtypes. While patients with sALS presented varied metabolic profiles, patients with fALS carrying *SOD1* mutations presented consistent decreases in glutamate and glutamine levels.^132^

Finally, recent studies have examined the neurochemical signature within the CSF of individuals with genetic forms of ALS. Specifically, compared with both patients with sALS and other patients with fALS, patients with ALS homozygous for the D90A *SOD1* mutation presented a distinct metabolic profile. Notably, these patients presented decreased levels of amino acids and their derivatives, including lysine, ornithine, serine, threonine and pyroglutamic acid, which appeared as distinct metabolic entities. Furthermore, this metabolomic profile remained unchanged in patients treated with riluzole.^132^

Gautam *et al.* investigated the potential metabolomic dysregulation potentially present in patients with ALS with TDP-43 pathology. Disruption of the NAD+/NADH ratio indicates energy deficiency and is associated with several pathologies, including ALS. Consistent with this notion, reduced levels of NAD+ were identified in the patient cohort. Because nicotinamide mononucleotide was reported to increase NAD+ levels, transgenic mice overexpressing mutant TDP-43 were treated with drugs targeting diseased corticospinal MNs. The results revealed that metabolic defects occur early in the motor cortex of patients with ALS and that restoring the NAD+ balance could offer therapeutic benefits to corticospinal MNs with TDP-43 pathology.^7^

#### *In vivo* studies

Different studies have been performed using rodent models to investigate the metabolome in individuals with ALS. Several lines of evidence suggest that metabolic changes may precede symptoms in SOD1-G93A ALS mouse models. *In vivo* H-NMR revealed that, compared with wild-type mice, transgenic SOD1 mice presented increased cortical levels of glutamate (Glu). Notably, the administration of creatinine reduced Glu levels and slowed disease progression in mice with ALS.^133^ Nevertheless, a clinical trial on patients with ALS treated with creatinine did not show any clinical benefits in humans.^134^

Another study on various CNS tissues of SOD1-G93A mice via high-resolution H-MRS revealed reduced concentrations of NAA, Gln and GABA in the brainstem and spinal cord. Notably, these changes appeared earlier in the spinal cord and later in brainstem, before signs of MN degeneration might be identified. This spatial progression pattern of metabolic changes suggested retrograde degeneration, with NAA as a potential candidate biomarker.^135^

The effect of creatine supplementation on ALS progression was assessed in the SOD1-G93A mouse model and *in vitro* via H-NMR spectroscopy.^136^  *In vitro*, the spinal cord presented the most abnormalities, with reduced NAA and N-acetylaspartylglutamate levels and increased Glu, Tau and inositol levels. Similarly, the sensorimotor cortex displayed increased Glu and Gln, and the brainstem presented increased concentrations of Glu, Gln, glycine (Gly) and taurine. For *in vivo* studies, transgenic mice were divided into three groups: ‘unaffected’, ‘paralysis of the hindlimbs’ and ‘paralysis of hind limbs with partial involvement of the forelimbs’. Compared with the unaffected group, the hind limb paralysis group presented increased NAA/Cr ratios in the motor cortex. Dietary supplementation with Cr was able to partially rescue NAA loss.^136^

Depending on the background mouse strain used, the progression of the phenotype in mice expressing SOD1-G93A can vary widely. Recently, Valbuena’s group compared the metabolic profiles of the spinal cord of transgenic mice with rapidly progressing and slowly progressing ALS via GC–MS. Both the lumbar and the thoracic spinal cord tracts presented tissue metabolomic differences. The affected spinal cord presented alterations in metabolites involved in energy and lipid metabolism. The thoracic tracts presented more consistent presymptomatic alterations in metabolites involved in energy homeostasis, neurotransmitter synthesis and the oxidative stress response. In addition, mice with rapidly progressing ALS presented greater metabolic impairments, likely conferring a disadvantage in maintaining neuronal survival.^137^

Irisin, a myokine secreted by both skeletal muscle and the brain, was recently studied in myosin light chain MLC/SOD1-G93A mice. Its plasma concentration increases after skeletal muscle activity and stimulates the synthesis of brain-derived neurotrophic factor in the nervous system, which has a neuroprotective function, thus indicating a possible link between muscle and the CNS. In this study, MLC/SOD1-G93A transgenic mice presented lower expression of irisin than control mice did, suggesting that this reduction may affect neuromuscular junctions.^138^

The ability of nicotinamide riboside, a precursor of NAD+, to increase its bioavailability in mice with ALS was analysed. A slowing of neuronal degeneration and overall increased survival were reported in treated SOD1 mice. These findings suggest the need for further testing of NAD+ supplementation in humans.^139^

#### In vitro studies

*In vitro* models have become essential tools for studying the metabolic disruptions associated with ALS, providing crucial insights into disease mechanisms and potential therapeutic targets. Studies using iPSC-derived MNs have revealed significant alterations in energy metabolism and mitochondrial function characteristic of ALS pathology.^140^ The integration of metabolomics with other molecular approaches has uncovered critical disruptions in cellular bioenergetics, with specific emphasis on altered glucose metabolism and mitochondrial dysfunction in MNs derived from patients with ALS.^141^ Other metabolomic analyses of ALS *in vitro* models have identified disruptions in glycolytic pathways and increased lactate production, indicating a shift towards aerobic glycolysis. These metabolic alterations suggest a reprogramming of energy production mechanisms in ALS MNs, contributing to disease progression.^142^

Additional studies employing NSC-34 and astrocyte co-cultures have demonstrated how oxidative stress, induced by compounds such as menadione, exacerbates mitochondrial dysfunction and glutamate excitotoxicity. LC–MS and GC–MS analyses revealed reductions in tricarboxylic acid (TCA) cycle intermediates such as succinate, citrate and malate, alongside elevated extracellular glutamate levels due to astrocyte dysfunction. This interplay between impaired astrocytic glutamate recycling and mitochondrial energy deficits accelerates neuronal degeneration, reinforcing the importance of targeting both pathways for therapeutic intervention.^143^

NMR spectroscopy has also proven invaluable for studying blood–brain barrier (BBB) dysfunction in ALS, enabling the detection of metabolite shifts in *in vitro* BBB models. These studies highlight how oxidative stress-induced glutamate dysregulation compromises BBB integrity, contributing to increased permeability and neurodegeneration. Alterations in lipid metabolites, crucial for maintaining cell membranes and tight junctions, further elucidate the metabolic vulnerabilities of the BBB under pathological conditions. In astrocytic models, targeted metabolomics has revealed deficits in energy metabolism, particularly in lactate shuttling.^144^ Reduced lactate levels, coupled with downregulation of key transport proteins such as SLC16A4 and GLAST-1, result in neuronal energy deficits and exacerbated ionic imbalances. These findings highlight the pivotal role of astrocytes in sustaining neuronal metabolic homeostasis.^145^

Additionally, metabolic profiling of fibroblasts and induced astrocytes derived from patients with ALS has shed light on age-related metabolic dysfunctions. Phenotypic metabolic microarrays revealed impaired glycolytic pathways and reduced metabolism of key energy substrates, such as glycogen and lactic acid, which are crucial for cellular adaptation to aging. Metabolic flux analysis confirmed deficits in mitochondrial respiration and glycolytic capacity, linked to enzymatic deficiencies in glycogen metabolism. Interestingly, supplementation with inosine or α-ketoglutaric acid enhanced metabolic efficiency in these cells, suggesting potential therapeutic interventions.^146^ Furthermore, studies on MNs differentiated from ALS patient-derived iPSCs revealed metabolic reprogramming during differentiation, characterized by increased lactate oxidation and enhanced TCA cycle activity. Notably, ALS-associated *FUS* mutations did not significantly impact these metabolic pathways *in vitro*, suggesting that energy metabolism dysfunction may not be the primary driver of ALS in this context.^147^

Collectively, these studies underscore the value of *in vitro* metabolomics in dissecting ALS pathology. By elucidating the interconnected roles of glutamate excitotoxicity, mitochondrial dysfunction, astrocyte–neuron interactions and BBB integrity, these models provide a comprehensive framework for identifying biomarkers and developing targeted therapeutic strategies. Advanced metabolomic platforms, including LC–MS, GC–MS and NMR, offer robust tools for real-time tracking of metabolic changes, bridging the gap between *in vitro* findings and clinical applications. These insights pave the way for precision medicine approaches aimed at mitigating ALS progression and improving patient outcomes.

### Microbiomics

Microbiomics, the study of microbial communities and their genetic compositions, has emerged as a powerful tool for elucidating the intricate interplay between microbial dysbiosis and disease. Unravelling the genetic blueprints of microbial populations enables the identification of microbial signatures associated with specific diseases, providing valuable diagnostic and prognostic markers.

The mechanisms underlying gut microbiota dysbiosis in individuals with ALS are multifaceted and may involve alterations in immune responses, gut barrier integrity and microbial metabolite production. Dysbiotic microbiota may induce immune dysregulation and neuroinflammation through the activation of toll-like receptors and the production of proinflammatory cytokines.^148^ Furthermore, impaired gut barrier function, characterized by increased intestinal permeability, may facilitate the translocation of microbial products and systemic inflammation.^149^ Additionally, alterations in the production of microbial metabolites, such as short-chain fatty acids and neurotoxic metabolites, may contribute to the pathogenesis of ALS.^150^ The gut microbiome is a source of bioactive metabolites that affect synaptic plasticity, myelination and host behaviours, potentially contributing to modifying disease progression.^151-153^

An analysis of the ALS gut microbiome has produced controversial results. Although some studies have reported no alterations in the microbiome of patients with ALS,^154^ others have demonstrated dysbiosis, characterized by reduced microbial biodiversity, which is frequently associated with gut inflammation.^155,156^ In particular, patients present an alteration in the Firmicutes/Bacteroidetes ratio, a condition that is correlated with several human gut disorders.^156^ In addition, stool samples presented altered metabolomes, with increased levels of propanoic acid and coproporphyrinogen and reduced acylcarnitine.^157^

Intestinal bacteria may also contribute to the pathogenesis of ALS via the generation of neurotoxins. In fact, specific strains of *Clostridium* are known to produce neurotoxins that specifically target the motor system. However, although strains such as *Clostridium baratii* and *Clostridium butyricum* are known to produce neurotoxins, their precise neurological effects remain unclear. The colonization of patients with ALS with specific species of neurotoxin-producing bacteria within the MN environment may impact disease progression in susceptible individuals.^150^ These findings further support the potential role of the microbiome in the progression of MND and ALS.

Building upon previous discussions that highlighted alterations in microbiome composition among individuals with ALS, researchers have extended these observations to rodent models, where similar changes have been observed and suggested to determine dysbiosis-induced alterations in metabolic pathways.^149,158,159^ Notably, SOD1 transgenic mice exhibited presymptomatic dysbiosis coupled with metabolic modifications that exacerbated the progression of the disease.^159^ The administration of specific bacteria, such as *Ruminococcus torques* and *Parabacteroides distasonis*, appeared to aggravate disease signs in mouse models, whereas interventions involving commensal bacteria, such as *Akkermansia muciniphila* (AM), have shown promise in alleviating the disease phenotype.^152,159^ The protective mechanism underlying the AM effect might involve AM-induced accumulation of nicotinamide in the CNS, as further confirmed by systemic supplementation in SOD1 mice. Specifically, nicotinamide has demonstrated efficacy in ameliorating motor symptoms and gene expression patterns in the spinal cord of transgenic ALS models. These results suggest that microbiome-derived metabolites could plausibly translocate to the CNS and affect disease progression in ALS mouse models.

Taken together, these comprehensive investigations, ranging from human research to experimentation in rodent models, emphasize the pivotal role of microbiome-derived metabolites in shaping the course of ALS pathology. These findings provide valuable insights into potential therapeutic strategies while highlighting the complex interplay between the gut microbiota and CNS function.

### Lipidomics

Early lipidomic studies were based on indirect techniques, which, owing to their low specificity, were limited by detecting only lipid molecules present in high abundance in serum, such as cholesterol and triglycerides. The advent of new molecular investigation techniques, such as MS, has made it possible to identify all lipid species present in serum with high precision and efficiency, enabling the detection of even minute variations in the concentration of specific lipids crucial for disease progression.

Intriguingly, in the last decade, several dysregulated lipid-related pathways have been reported to contribute to ALS pathogenesis and development. Indeed, lipid-specific metabolic abnormalities might precede the development of motor symptoms in individuals with ALS and correlate with disease progression. In fact, presymptomatic ALS mouse models exhibit impaired lipid pathways, indicating the potential of lipids as predictive biomarkers.^160^ In addition, both patients with ALS and animal models have longer survival rates and slower disease progression when they express higher concentrations of total cholesterol, low-density lipoprotein (LDL) levels, LDL/high-density lipoprotein ratio and triglycerides, possibly indicating an association between lipid homeostasis and disease prognosis.^160-162^ Among these metabolites, diglycerides, triglycerides and cholesterol esters have been proposed as candidate biomarkers, as have apolipoprotein B and LDL.^163,164^ However, the relationship between lipid metabolism and ALS remains uncertain, and a clear correlation between dyslipidemia and the incidence of ALS is still lacking.^162^

Indeed, studies on human serum and CSF have yielded mixed results. Fernández-Eulate *et al*. performed unbiased lipidomic profiling of patient serum via ultrahigh-performance LC-MS (UPLC-MS). Although patients with ALS presented altered triacylglycerol and oxidized fatty acid levels compared with those in the control cohort, these differences were not significant when adjusted for multiple comparisons. Specific monounsaturated fatty acid, triglyceride and sphingomyelin molecules further discriminate between patients with ALS and controls.^165^ Another study evaluated the CSF lipidomic signature in patients with ALS compared with healthy controls. Patients with ALS present a specific lipidomic profile, with significantly different levels of phosphatidylcholine, ceramides and glucosylceramides. These results were confirmed via neuropathological analysis of the brain cortex of ALS model mice. In parallel, the authors generated models for predicting clinical evolution (measured with the ALSFRS-R score) from the baseline lipidome, with an accuracy of 71%.^166^

Using a multiomics approach, Lee *et al*.^167^ were able to identify an imbalance in lipid metabolism, specifically regarding the arachidonic acid (AA) pathway. For this reason, both *in vivo* and *in vitro* models were treated pharmacologically to rebalance AA levels, which were found to be altered in patients with ALS. This treatment resulted in an amelioration of the phenotypes, suggesting that pharmacological modulation of the AA pathway may have a protective effect.^167^

Recently, Chaves-Filho *et al*. performed an untargeted MS-based lipidomic analysis of the motor cortex and the spinal cord of a rat model overexpressing the human mutant SOD1-G93A in comparison with wild-type mice during both the presymptomatic and symptomatic stages of the disease. While the lipidome profile in the motor cortex displayed age-related alterations in both groups, the spinal cord showed consistent lipidome disruption, particularly in symptomatic mice. These changes included decreased levels of cardiolipin and increased concentrations of different cholesteryl esters associated with polyunsaturated fatty acids.^168^

Overall, these findings in ALS animal models and humans highlight the potential role of lipidomic analysis in elucidating aspects of ALS pathophysiology. These findings suggest that further exploration of lipidomic profiles could provide valuable insights into the mechanisms underlying ALS progression and aid in the development of novel therapeutic approaches aimed at addressing lipid-related alterations in this disease.

## Conclusion

The multiple alterations underlying ALS pathogenesis and the current lack of effective ALS therapy suggest that successful treatment will require the targeting of multiple pathways, especially in those cases lacking a known causative genetic mutation.^29^

In the last decade, omics approaches have yielded invaluable insights into ALS pathogenesis. In particular, proteomics has led to the identification of disease-related proteins in biofluids that might represent reliable sources of candidate biomarkers. For example, a shorter life expectancy and more rapid disease progression have been associated with higher levels of NFL and p-NFH. Another protein with good potential is pTau, which may discriminate ALS from ALS-mimic syndromes and other neurodegenerative diseases, although with less accuracy than NFs.^59^ Furthermore, SOD1 protein levels in the CSF of patients with ALS with mutations in *SOD1* have been employed as a pharmacodynamic biomarker to quantify target engagement in clinical trials with *SOD1*-directed ASOs,^35,37^ whereas alterations in poly(GP) levels in patients with *C9ORF72*-ALS seem to negatively correlate with the amount of HRE.^27,33^ Several other studies revealed that multiple proteins are intertwined in many key cellular processes, reflecting the disruption of the cellular systems fundamental for cell survival. Taken together, these findings suggest that proteomic studies carried out in biofluids from patients with ALS and disease models may help in the discovery of novel pathogenic targets and the identification of disease biomarkers in a non-invasive manner.

Despite these significant advancements, the application of omics approaches to ALS research still faces several challenges and open questions. One major barrier is the heterogeneity in data integration and interpretation, as large-scale datasets require complex bioinformatic pipelines and standardization across cohorts and platforms. Reproducibility is often hampered by variability in experimental protocols, sample types and analytical methods. Furthermore, distinguishing disease-specific changes from those related to comorbidities or aging processes remains a significant hurdle, emphasizing the need for rigorous validation of findings in independent cohorts and disease models.

The high costs, technical complexity and requirement for specialized expertise also limit the widespread implementation of omics in the clinical practice. Additionally, the integration of multiomics data for a holistic understanding of ALS requires advanced computational tools capable of handling diverse datasets, alongside addressing ethical considerations such as data privacy and equitable access to these technologies.

Nonetheless, omics approaches have demonstrated considerable strength in uncovering novel molecular insights and stratifying patients into distinct subgroups, paving the way for personalized medicine. Findings from metabolomics and microbiomics, for instance, have revealed novel perspectives on metabolic dysregulation and the gut–brain axis in ALS, providing opportunities for innovative therapeutic strategies. Metabolomic studies revealed neurochemical alterations that might be helpful in the longitudinal monitoring of ALS disease progression and response to treatment. Plasma from patients with ALS presented elevated metabolites associated with hypermetabolic processes, oxidative changes and mitochondrial dysfunction. Fluid metabolite profiling might also facilitate the differential diagnosis of ALS, distinguishing between different phenotypes and correlating with disease severity.

The FDA recently approved tofersen mostly on the basis of its NFL-lowering effect, but whether *SOD1*-directed ASOs represent a disease-modifying therapy for ALS still needs to be clarified.^37,169^ However, NFL assessment is becoming an established practice among clinical trials, and its widespread routine clinical use should be encouraged to understand its potential as a sensitive marker of treatment efficacy and an early indicator of neurodegenerative diseases.^169^ Nonetheless, a standardized and globally accessible method for NFL measurement is needed to fully incorporate this biomarker into clinical practice.^170^

The same approach should be applied to specific biomarkers associated with genetic forms of ALS. Since an ASO-based therapy has recently been approved for *SOD1*-ALS, genetic testing for this form should be mandatory. Moreover, because SOD1 and NFL levels in CSF exhibit similar behaviour, SOD1 measurement also needs to be encouraged.^37^

DPRs might represent CSF biomarkers specific for *C9ORF72*-ALS. Although their levels do not seem to correlate with disease onset, severity and progression, data from preclinical models and preliminary clinical data suggest the potential use of DPRs as pharmacodynamic biomarkers, as their CSF concentrations are reduced by ASO treatment.^27,34,42,90^ Given their limited application in clinical practice, DPR assessments should be performed in clinical trials to monitor treatment effects. However, data from animal models might have limited translatability.

Despite promising results and accumulating knowledge on ALS pathomechanisms, to date, a specific disease-modifying treatment is still not available. In fact, the majority of available biomarkers seem to be associated with advanced stages of disease when a therapeutic intervention might be less effective. Therefore, early-onset biomarkers that can predict disease onset are urgently needed. The use of standardized validated assays should also be encouraged. The heterogeneity of ALS clinical manifestations indicates the concomitance of multiple dysregulations, eventually resulting in neurodegeneration. Omics has improved our way of approaching ALS, shedding light on the complex molecular pathomechanisms of the disease and identifying different patient subtypes, thus providing hints for the development of new individualized treatments. In particular, proteomics provides reliable candidate biomarkers for differential diagnosis and treatment follow-up. Greater integration of proteomics results, together with technological advances in newer platforms, could provide better insights into ALS disease pathogenesis, support patient stratification and prognosis and drive the development of specific therapies.

## Supplementary Material

fcaf114_Supplementary_Data

## Data Availability

Data sharing is not applicable to this article as no new data were created or analysed in this study.
