# Supplementary material for: Unveiling amyotrophic lateral sclerosis complexity: insights from proteomics, metabolomics and microbiomics
Source: Brain Commun. 2025 Mar 19;7(2):fcaf114. doi: 10.1093/braincomms/fcaf114 (PMC11952287; doi:10.1093/braincomms/fcaf114)
Supplement: fcaf114_Supplementary_Data [file fcaf114_supplementary_data.docx]

**Supplementary Table 1. Findings from proteomics studies in amyotrophic lateral sclerosis grouped by model.**

| **Model** | **Tissue sample** | **ALS subgroup** | **Protein** | ↑/↓ | **Pathway** | **Biological relevance** | **References** |
| --- | --- | --- | --- | --- | --- | --- | --- |
| Human | CSF | C9ORF72 | Poly(GP) | ↑ | DPRs toxicity | Correlation with expansions amount | 1, 2 |
| Human | CSF | SOD1 | CYPA | ↓ | Proteostasis, BH4 homeostasis, mitochondrial ATP production, oxidative stress | ALS susceptibility | 3 |
| Human | CSF | SOD1 | HSP90α | ↓ | Proteostasis | ALS susceptibility | 3 |
| Human | CSF | SOD1 | BiP | ↓ | Proteostasis | ALS susceptibility | 3 |
| Human | CSF | SOD1 | DJ-1 | ↓ | Proteostasis | ALS susceptibility | 3 |
| Human | CSF | sALS, fALS | EEF1A1 | ↓ | DNA or RNA binding proteins involved in transcription and translation, TDP43 interacting protein |  | 4 |
| Human | CSF | sALS, fALS | H2BC11 | ↓ | DNA or RNA binding proteins involved in transcription and translation, TDP43 interacting protein |  | 4 |
| Human | CSF | sALS, fALS | ANP32A | ↑ | DNA or RNA binding proteins involved in transcription and translation |  | 4 |
| Human | CSF | sALS, fALS | YBX1 | ↓ | DNA or RNA binding proteins involved in transcription and translation, TDP43 interacting protein |  | 4 |
| Human | CSF | sALS, fALS | TUBB | ↓ | Microtubule associated protein, TDP43 interacting protein |  | 4 |
| Human | CSF | sALS, fALS | GAPDH | ↓ | Glycolysis |  | 4 |
| Human | CSF | sALS, fALS | LDHA | ↓ | Glycolysis and anaerobic metabolic pathway |  | 4 |
| Human | CSF | sALS, fALS | MIF | ↓ | Innate immunity |  | 4 |
| Human | CSF | sALS, fALS | NRXN1 | ↓ | Synaptic protein |  | 4 |
| Human | CSF | sALS, fALS | NRXN3 | ↓ | Synaptic protein |  | 4 |
| Human | CSF | sALS, fALS | EPHA4 | ↓ | Neural growth factor | Correlate with severity of disease, earlier onset but slower progression | 4 |
| Human | CSF | sALS, fALS | CDH13 | ↓ | Guidance protein and cell adhesion molecule |  | 4 |
| Human | CSF | sALS, fALS | CHIT1 | ↑ | Neuroinflammation: microglial activity marker | Diagnosis and progression | 1, 4, 5 |
| Human | CSF | sALS, fALS | CHI3L1 | ↑ | Neuroinflammation: microglial activity marker | Diagnostic | 4, 5 |
| Human | CSF | sALS, fALS | CHI3L2  YKL39 | ↑ | Neuroinflammation: microglial activity marker | Diagnostic | 4, 5 |
| Human | CSF | sALS, fALS | MAP2 | ↑ | Cytoskeletal component |  | 6 |
| Human | CSF | sALS, fALS | HIST1H4A | ↓ | Histone protein |  | 6 |
| Human | CSF | sALS, fALS | HIST1H2B | ↓ | Histone protein |  | 6 |
| Human | CSF | sALS, fALS | GMCSF | ↑ | Neuroinflammation: immune response | Diagnostic | 7 |
| Human | CSF | sALS, fALS | IL-2 | ↑ | Neuroinflammation: immune response | Diagnostic | 7 |
| Human | CSF | sALS, fALS | MIP-1α | ↑ | Chemotactic chemokines | Prognostic | 1 |
| Human | CSF | sALS, fALS | wrCRP | ↑ | Immune response | Prognostic: more rapidly progressive disease | 1 |
| Human | CSF | sALS, fALS | HMGB | ↑ | Chromatin protein: interact with nucleosomes, transcription factors and histones | Prognostic: more rapidly progressive disease | 1 |
| Human | CSF | sALS, fALS | CPK | ↑ | Muscular contraction | Prognostic: more rapidly progressive disease | 1 |
| Human | CSF | sALS, fALS | GrB | ↑ | Inflammatory response | Prognostic: more rapidly progressive disease | 1 |
| Human | CSF | sALS, fALS | IL-8 | ↑ | Inflammatory response | Prognostic | 1 |
| Human | CSF | sALS, fALS | CST3 | ↑ | Inhibitor of lysosomal proteinases | Prognostic: positively correlated with survival | 1, 8 |
| Human | CSF | sALS, fALS | GPNMB | ↑ | Inflammatory response | Prognostic: short survival time | 1 |
| Human | CSF | sALS, fALS | bFGF, FGF2, FGFβ | ↑ | Growth factor | Prognostic: longer survival | 1 |
| Human | CSF | sALS, fALS | VGF | ↑ | Nerve growth, synaptic plasticity | Prognostic: progressive muscle weakness | 1 |
| Human | CSF, Plasma | sALS, fALS | NFs | ↑ | Cytoskeletal components | Diagnostic, Prognostic | 7, 9 |
| Human | CSF, Plasma | sALS, fALS | TDP43, pTDP43 | ↑ | RNA metabolism | Diagnostic, Prognostic | 10, 11, 12 |
| Human | CSF, Plasma | sALS, fALS | tTau, pTau | ↓ | Microtubule associated protein | Diagnostic, Prognostic, Severity | 1, 13, 14, 15 |
| Human | CSF, Plasma | SOD1 | SOD1 | ↑ | Oxidative stress | Diagnostic | 1, 16, 17 |
| Human | CSF, Plasma | sALS, fALS | IL-10 | ↓ | Neuroinflammation: immune response | Diagnostic | 7 |
| Human | CSF, Plasma | sALS, fALS | IL-6 | ↓ | Neuroinflammation: immune response | Diagnostic | 7 |
| Human | CSF, Plasma | sALS, fALS | IL-15 | ↓ | Neuroinflammation: immune response | Diagnostic | 7 |
| Human | CSF,  Spinal cord | sALS, fALS | UCHL1 | ↑ | Correct function of the ubiquitin-proteasome-system |  | 1, 6 |
| Human | CSF,  Spinal cord | sALS, fALS | CAPG | ↑ | Inflammatory processes |  | 6 |
| Human | CSF,  Spinal cord | sALS, fALS | GPNMB | ↑ | Inflammatory processes | Prognostic: shorter life expectancies and disease severity | 6 |
| Human | CSF,  Spinal cord | sALS, fALS | RBM45 | ↓ | Precipitate and form intracytoplasmic inclusion |  | 18 |
| Human | Fibroblasts | sALS, fALS | ApoB48 | ↓ | Fats metabolism |  | 19 |
| Human | Fibroblasts | sALS, fALS | HSP20 | ↓ | Response to environmental stress |  | 19 |
| Human | Fibroblasts | sALS, fALS | FBLN1 | ↑ | Cell adhesion and migration |  | 19 |
| Human | iMNs | C9ORF72 | Poly(GP) | ↑ | DPRs toxicity | Pharmacodynamic marker after ASO treatment | 1, 20 |
| Human | Plasma | sALS, fALS | ApoE | ↑ | Fats metabolism | Prognostic: rapid progression, short survival | 21 |
| Human | Spinal cord | sALS, fALS | HNRNPC | ↑ | RNA splicing |  | 6 |
| Human | Spinal cord | sALS, fALS | SNRPNB2 | ↑ | RNA splicing |  | 6 |
| Human | Spinal cord | sALS, fALS | MAGOH | ↑ | RNA splicing |  | 6 |
| Human | Spinal cord | sALS, fALS | SNRPE | ↑ | RNA splicing |  | 6 |
| Human | Spinal cord | sALS, fALS | LSM3 | ↑ | RNA splicing |  | 6 |
| Human | Spinal cord | sALS, fALS | SNRPD2 | ↑ | RNA splicing |  | 6 |
| Human | Spinal cord | sALS, fALS | SRSF1 | ↑ | RNA splicing |  | 6 |
| Human | Spinal cord | sALS, fALS | LSM7 | ↑ | RNA splicing |  | 6 |
| G93A mice | Spinal cord homogeneates | SOD1-ALS | SOD1 | ↑ | Oxidative stress | Pharmacodynamic marker after ASO treatment | 16, 17 |
| Human | Fibroblasts | C9ORF72-ALS | RAN | ↓ | translocation of RNA and proteins through the NPC |  | 22 |
| Human | Fibroblasts | C9ORF72-ALS | RAN-GAPs | ↓ | translocation of RNA and proteins through the NPC |  | 22 |
| Human | Fibroblasts | C9ORF72-ALS | VPS4B | ↓ | vesicle trafficking |  | 22 |
| Human | Fibroblasts | C9ORF72-ALS | COX2 | ↓ | ATP synthesis |  | 22 |
| Human | Fibroblasts | C9ORF72-ALS | COX5A | ↓ | ATP synthesis |  | 22 |
| Human | Fibroblasts | C9ORF72-ALS | VDAC1 | ↓ | ATP synthesis |  | 22 |
| Human | Fibroblasts | C9ORF72-ALS | STAT1 | ↓ | transcription factor |  | 22 |

**Abbreviations**: ANP32A: Acidic leucine-rich nuclear phosphoprotein 32 family member A; ApoB48: Apolipoprotein B48; ApoE: Apolipoprotein E; bFGF/ FGF2/ FGFβ: basic fibroblast growth factor; BiP: Binding immunoglobulin protein; CAPG: Capping actin protein, gelosin-like; CDH13: Cadherin 13 precursor; CHI3L1: Chitinase-3-like protein 1; CHI3L2/YKL39 : Chitinase-3-like protein 2; CHIT: Chitotriosidase 1; CPK: Creatinine kinase; CST3: Cystatin C; CYPA: Cyclophilin A; DJ-1: Deglycase protein; EEF1A1: Putative elongation factor 1-alpha 1; EPHA4: Ephrin type A receptor 4; FBLN1; Fibulin-1; GAPDH: Glyceraldehyde-3-phosphate dehydrogenase; GMCSF: Granulocyte macrophage colony stimulating factor; GPNMB: Glycoprotein Nmb; GPNMB: Glycoprotein non-metastatic melanoma protein B; GrB: Granzyme B; H2BC11: Histone H2B type 1-N; HIST1H2B: Histone H2B type 1-B; HIST1H4A: Histone H4; HMGB: High mobility group box / Amphoterin; HNRNPC: Heterogeneous nuclear ribonucleoproteins C1/C2; HSP20: Heat shock protein 20; HSP90α: Heat-shock protein 90α; IL-10: Interleukine 10; IL-15: Interleukine 15; IL-2: Interleukine 2; IL-6: Interleukine 6; IL-8: Interleukine 8; LDHA: Lactate dehydrogenase; LSM3: LSM3 Homolog / U6 Small Nuclear RNA And mRNA Degradation Associated; LSM7: U6 snRNA-associated Sm-like protein LSm7; MAGOH: Protein mago nashi homolog; MAP2: Microtuble associated protein 2; MIF: Macrophage migration inhibitory factor;

MIP-1α: Macrophage Inflammatory Protein-1 Alpha; NFs: Neurofilaments; NRXN1: Neurexin 1; NRXN3: Neurexin 3; Poly(GP): Poly Glycine-Proline dipeptide repeats proteins; RBM45: RNA-binding motif 45 protein; SNRPD2: Small Nuclear Ribonucleoprotein D2 Polypeptide; SNRPE: Small nuclear ribonucleoprotein E; SNRPNB2: Small nuclear ribonucleoprotein polypeptide N B2; SOD1: Superoxide dysmutase 1; SRSF1: Serine/arginine-rich splicing factor 1; TDP-43, pTDP-43: TAR DNA binding protein 43, phosphorylated TDP-43; tTau, pTau: total and Phosphorylated Tau; TUBB: Tubulin beta chain; UCHL1: Ubiquitin C-terminal hydrolase-L1; VGF: Nerve growth factor inducibile VGF; wrCRP: C Reactive Protein; YBX1: Y-box-binding protein 1.

**Supplementary references**

1. Verber N, Shaw PJ. Biomarkers in amyotrophic lateral sclerosis: a review of new developments. *Curr Opin Neurol*. 2020;33(5):662-668. doi:10.1097/WCO.0000000000000854

2. Cammack AJ, Atassi N, Hyman T, et al. Prospective natural history study of C9orf72 ALS clinical characteristics and biomarkers. *Neurology*. 2019;93(17):e1605-e1617. doi:10.1212/WNL.0000000000008359

3. Filareti M, Luotti S, Pasetto L, et al. Decreased Levels of Foldase and Chaperone Proteins Are Associated with an Early-Onset Amyotrophic Lateral Sclerosis. *Front Mol Neurosci*. 2017;10:99. doi:10.3389/fnmol.2017.00099

4. Thompson AG, Gray E, Charles PD, et al. Network Analysis of the CSF Proteome Characterizes Convergent Pathways of Cellular Dysfunction in ALS. *Frontiers in Neuroscience*. 2021;15. Accessed May 9, 2022. <https://www.frontiersin.org/article/10.3389/fnins.2021.642324>

5. Thompson AG, Gray E, Thézénas ML, et al. Cerebrospinal fluid macrophage biomarkers in amyotrophic lateral sclerosis. *Ann Neurol*. 2018;83(2):258-268. doi:10.1002/ana.25143

6. Oeckl P, Weydt P, Thal DR, Weishaupt JH, Ludolph AC, Otto M. Proteomics in cerebrospinal fluid and spinal cord suggests UCHL1, MAP2 and GPNMB as biomarkers and underpins importance of transcriptional pathways in amyotrophic lateral sclerosis. *Acta Neuropathol*. 2020;139(1):119-134. doi:10.1007/s00401-019-02093-x

7. Morello G, Salomone S, D’Agata V, Conforti FL, Cavallaro S. From Multi-Omics Approaches to Precision Medicine in Amyotrophic Lateral Sclerosis. *Frontiers in Neuroscience*. 2020;14. Accessed May 9, 2022. <https://www.frontiersin.org/article/10.3389/fnins.2020.577755>

8. Raghunathan R, Turajane K, Wong LC. Biomarkers in Neurodegenerative Diseases: Proteomics Spotlight on ALS and Parkinson’s Disease. *International Journal of Molecular Sciences*. 2022;23(16):9299. doi:10.3390/ijms23169299

9. Gagliardi D, Meneri M, Saccomanno D, Bresolin N, Comi GP, Corti S. Diagnostic and Prognostic Role of Blood and Cerebrospinal Fluid and Blood Neurofilaments in Amyotrophic Lateral Sclerosis: A Review of the Literature. *Int J Mol Sci*. 2019;20(17):E4152. doi:10.3390/ijms20174152

10. Majumder V, Gregory JM, Barria MA, Green A, Pal S. TDP-43 as a potential biomarker for amyotrophic lateral sclerosis: a systematic review and meta-analysis. *BMC Neurol*. 2018;18(1):90. doi:10.1186/s12883-018-1091-7

11. Ren Y, Li S, Chen S, et al. TDP-43 and Phosphorylated TDP-43 Levels in Paired Plasma and CSF Samples in Amyotrophic Lateral Sclerosis. *Frontiers in Neurology*. 2021;12. Accessed May 9, 2022. https://www.frontiersin.org/article/10.3389/fneur.2021.663637

12. Beyer L, Günther R, Koch JC, et al. TDP-43 as structure-based biomarker in amyotrophic lateral sclerosis. *Ann Clin Transl Neurol*. 2021;8(1):271-277. doi:10.1002/acn3.51256

13. Steinacker P, Feneberg E, Weishaupt J, et al. Neurofilaments in the diagnosis of motoneuron diseases: a prospective study on 455 patients. *J Neurol Neurosurg Psychiatry*. 2016;87(1):12-20. doi:10.1136/jnnp-2015-311387

14. Scarafino A, D’Errico E, Introna A, et al. Diagnostic and prognostic power of CSF Tau in amyotrophic lateral sclerosis. *J Neurol*. 2018;265(10):2353-2362. doi:10.1007/s00415-018-9008-3

15. Grossman M, Elman L, McCluskey L, et al. Phosphorylated Tau as a Candidate Biomarker for Amyotrophic Lateral Sclerosis. *JAMA Neurology*. 2014;71(4):442-448. doi:10.1001/jamaneurol.2013.6064

16. Une M, Yamakawa M, Watanabe Y, et al. SOD1-interacting proteins: Roles of aggregation cores and protein degradation systems. *Neurosci Res*. 2021;170:295-305. doi:10.1016/j.neures.2020.07.010

17. Gertsman I, Wuu J, McAlonis-Downes M, et al. An endogenous peptide marker differentiates SOD1 stability and facilitates pharmacodynamic monitoring in SOD1 amyotrophic lateral sclerosis. *JCI Insight*. 4(10):e122768. doi:10.1172/jci.insight.122768

18. Collins M, Riascos D, Kovalik T, et al. The RNA-binding motif 45 (RBM45) protein accumulates in inclusion bodies in amyotrophic lateral sclerosis (ALS) and frontotemporal lobar degeneration with TDP-43 inclusions (FTLD-TDP) patients. *Acta Neuropathol*. 2012;124(5):717-732. doi:10.1007/s00401-012-1045-x

19. Narayan M, Seeley KW, Jinwal UK. Identification of Apo B48 and other novel biomarkers in amyotrophic lateral sclerosis patient fibroblasts. *Biomark Med*. 2016;10(5):453-462. doi:10.2217/bmm-2016-0025

20. Gendron TF, Chew J, Stankowski JN, et al. Poly(GP) proteins are a useful pharmacodynamic marker for C9ORF72-associated amyotrophic lateral sclerosis. *Sci Transl Med*. 2017;9(383):eaai7866. doi:10.1126/scitranslmed.aai7866

21. Zubiri I, Lombardi V, Bremang M, et al. Tissue-enhanced plasma proteomic analysis for disease stratification in amyotrophic lateral sclerosis. *Molecular Neurodegeneration*. 2018;13(1):60. doi:10.1186/s13024-018-0292-2

22. Lualdi M, Shafique A, Pedrini E, et al. C9ORF72 Repeat Expansion Affects the Proteome of Primary Skin Fibroblasts in ALS. *Int J Mol Sci*. 2021;22(19):10385. doi:10.3390/ijms221910385
